# Bibliographical

**Published:** 1857-12

**Authors:** 


### Bibliographical.

We have received, from the publishers, through Messrs. J. J. Richards & Co., Booksellers, of this city, a number of valuable medical works, recently issued from the press of Messrs. Blanchard & Lea, of Philadelphia ; and first, we notice,

*Diseases of the Skin, by Erasmus Wilson, F. R. S., fourth American, from the fourth and enlarged London Edition,*

Containing 649 pages, and of a very comprehensive character, upon a most interesting, obscure, and little understood, but exceedingly important class of diseases. It comprehends the Anatomy and Physiology of the skin ; classification of diseases of the skin—General Pathology and General Therapeutics of the same structure, Erythematous or Exanthematous Eruptions, Lichenous or Papulous Eruptions, Eczematous or Vesicular Eruptions, Impetiginous or Pustular Eruptions, Herpetic and Bullous Eruptions, Furuncular Eruptions, and Scorbutic Eruption ; diseases arising from special internal causes, as Lepra Lupus, &c. ; diseases arising from special external causes, as Scabies, Malis, &c. ; diseases arising from the syphilitic poison ; diseases arising from animal poisons of unknown origin, and giving rise to eruptive fevers, as Rubeola, Scarlatina, &c. ; diseases affecting the special structure of the skin, embracing those of the vascular, nervous, papillary and pigmentary structures, with those of the sudoriferous and sebiparous organs; and, in addition, diseases affecting the hairs and hair follicles; nails and nail follicles, with a history and description of the Itch Animalcule, ("acarus scabiei") and of the Steatozoon Folliculorum, selected formulæ, and a very complete index. In reference to the present edition of this work, the author remarks: "I have arranged a new system of classification founded on the only true practical basis, the *cause* of the disease."

We doubt whether a better work upon the subject can be placed upon the table of the practitioner.

*Human Histology*, in its relations to Descriptive Anatomy, Physiology, and Pathology, with four hundred and thirty-four illustrations on wood, by E. R. Peaslee, A. M., M. D., Professor of Physiology and Pathology in the New York Medical College, of Anatomy in Dartmouth College, and of Surgery in the Medical School of Maine, member of the American Medical Association, &c., &c.,

"The aim of which is to give a connected view of the simple chemical elements, of the immediate principles, the simple structural elements and the proper tissues, entering into the composition of the fluids and the solids of the human body, and to associate with the structural elements and the tissues, their function while in health, and the changes they undergo in disease." "In regard to the dimensions of the minute structural elements, the work being intended especially for those who use the English language—they are given in fractions of an inch, instead of a millimetre, or a Paris line, as has usually been done by translators of German and French works, both in England and in this country."

From the examination we have been able to give the work, our impression of its merits is decidedly favorable. The subjects embraced are all important, and we agree with the author "that the only rational method of imparting a knowledge of *function* is by associating with the latter all that is known of the forms and relations of the structural elements, and of their chemical composition, in each part and organ." Pathological Anatomy and Pathology being placed in the same relation.

"*Principles of Medicine, an Elementary view of the causes, Nature, Treatment, Diagnosis and Prognosis of Disease, with brief remarks on Hygeinics or the Preservation of Health.* By CHARLES J. B. WILLIAMS, M. D., F. R. S. *A new American Edition from the third and revised London Edition.*"

This work, a new edition of which has been issued by Messrs. Blanchard & Lea, is so well known and so highly appreciated by the more scientific portion of the profession, that it may be considered standard, and it is only necessary to say, that considerable additions have been made to the present volume, bringing it up more fully to the present state of Physiology, General Pathology and Therapeutics, all of which it contains, to a very large extent. No Medical Library can be considered complete without it.

*Manual of Physiology.* By WILLIAM SENHOUSE KIRKES, M. D., Fellow of the Royal College of Physicians, &c., &c.

This is a new and revised American edition of the same Publishers, from the last London Edition, with two hundred illustrations, which has undergone a recent and careful revision at the hands of the author. This work having been selected by a number of Medical Colleges, as a text book, is perhaps the highest evidence which could be furnished as to its merits; and, to all, save those who desire an elaborate work, we would recommend it as the very best book on Physiology, with which we are acquainted, either for the student or practitioner.

We next find upon our table

"*The Effects of Climate on Tuberculous Dssease.* By Edwin Lee, M. R. C. S., London," and "*The Influence of Pregnancy on the Development of Tubercles.* By Edward Warren, M. D., of Edenton, N. C.,"

being two Essays combined in the shape of a handsome book. Though we have not yet been able to read them, our readers, we are sure, will consider us fully justified in commending them to their attention, when we state that "Dr. Caleb Fiske, who was President of the Rhode Island Medical Society in 1823 and 1824, at his death bequeathed to that Society a fund of two thousand dollars, directing the annual income to be expended in premiums for Essays on subjects selected for competition. The first premium of forty dollars was awarded June 27th, 1836, since which time a large number of valuable dissertations have been laid before the profession through the instrumentality of Dr. Fiske's well directed munificence. By the judicious management of the Trustees, the fund has gradually increased, and they are now able to offer two annual prizes of one hundred dollars each. *The Essays in the present volume are those to which the prizes of* 1855 *and* 1856 *were awarded.* Connected by the topics discussed, and presenting a large amount of important information on one of the most interesting and difficult subjects of medical science, the Trustees have been desirous of presenting them in a more permanent form, and they have been accordingly reprinted in their present shape from the *American Journal of the Medical Sciences*, for April and July, 1857, in which they originally appeared."

And lastly (though not least in value,) among the books received upon this occasion from these enterprising publishers, we come to notice

"*Lectures on the Diseases of Women.* By Charles West, M. D.," &c.,

Being, as the author remarks, a first instalment towards the discharge of that debt which the opportunities of a hospital and the responsibilities of a teacher have imposed upon him, promising within three years a second volume, which will treat of all the remaining diseases of the female system, this part being exclusively devoted to "Diseases of the Uterus." Embodying as this book does, the results of ten years' observation in the wards of a hospital, (St. Bartholomew's,) by a distinguished physician and accoucheur, it should at once command the attention of the profession, and we can assure those who may purchase it that they will not regret it. The author is clearly not a man of "one idea," or a contracted specialist, but of large and comprehensive mind, and much more practical than the most of those with whose plan of treating female diseases we have been familiar. The truth is, in our humble view, there has been and is a radical defect in treating diseases of this description, in many instances amounting to an apparently complete forgetfulness that the subject possessed any other organ than the Uterus, and an utter disregard of the most common and well understood physiological laws. As our author remarks, while absolute rest for a time is needed during the employment of the medical measures proper for the relief of uterine disease, it should never be forgotten that there are certain positive evils to which prolonged rest exposes a patient, both by the general interruption of her health from the confinement, and also by the almost inevitable direction of her thoughts, during the days of seclusion, from her ordinary pursuits and ordinary amusements, to the seat of suffering. The lectures embrace the Symptoms of the Diseases of Women—Menstruation and its various derangements; diseases of the Uterus proper; inflammation and ulceration; misplacements of the uterus, and the allied misplacements of vagina, bladder and rectum, their nature and mode of production; uterine tumours and outgrowths, with the treatment of these various departures from the state of health.

As previously intimated, the works which we have very imperfectly and briefly noticed, may be had of, or will be promptly ordered by, Messrs. J. J. Richards & Co., Booksellers, of this city, who keep constantly on hand a fine assortment of medical works.

In addition to the above, we have received the

*Transactions of the American Medical Association for the year* 1857,

Being the tenth volume, a copy of which every physician in the land should have in his library. The book can be obtained of Casper Wistar, M. D., of Philadelphia, by sending on $3.00.

